# Sirolimus and everolimus clearance in maintenance kidney and liver transplant recipients: Diagnostic efficiency of the concentration/dose ratio for the prediction of trough steady-state concentrations

**DOI:** 10.3109/03009730903291026

**Published:** 2010-04-07

**Authors:** Lorena Bouzas, Jesús Hermida, J. Carlos Tutor

**Affiliations:** Unidad Monitorización Fármacos, Laboratorio Central, Hospital Clínico Universitario, Santiago de CompostelaSpain

**Keywords:** Clearance, everolimus, predicted concentrations, sirolimus

## Abstract

**Objectives:**

Therapeutic monitoring of sirolimus and everolimus is necessary in order to minimize adverse side-effects and to ensure effective immunosuppression. A sirolimus-dosing model using the concentration/dose ratio has been previously proposed for kidney transplant patients, and the aim of our study was the evaluation of this single model for the prediction of trough sirolimus and everolimus concentrations.

**Methods:**

Trough steady-state sirolimus concentrations were determined in several blood samples from each of 7 kidney and 9 liver maintenance transplant recipients, and everolimus concentrations from 20 kidney, 17 liver, and 3 kidney/liver maintenance transplant recipients. Predicted sirolimus and everolimus concentrations (Css), corresponding to the doses (D), were calculated using the measured concentrations (Css_0_) and corresponding doses (D_0_) on starting the study: Css = (Css_0_)(D)/D_0_.

**Results:**

The diagnostic efficiency of the predicting model for the correct classification as subtherapeutic, therapeutic, and supratherapeutic values with respect to the experimentally obtained concentrations was 91.3% for sirolimus and 81.4% for everolimus in the kidney transplant patients. In the liver transplant patients the efficiency was 69.2% for sirolimus and 72.6% for everolimus, and in the kidney/liver transplant recipients the efficiency for everolimus was 67.9%.

**Conclusions:**

The model has an acceptable diagnostic efficiency (>80%) for the prediction of sirolimus and everolimus concentrations in kidney transplant recipients, but not in liver transplant recipients. However, considering the wide ranges found for the prediction error of sirolimus and everolimus concentrations, the clinical relevance of this dosing model is weak.

## Introduction

Sirolimus (Rapamune®, Wyeth Pharmaceuticals, Madison, NJ, USA) and everolimus (Certican®, Novartis Pharma AG, Basel, Switzerland), a derivative of sirolimus with a 2-hydroxyethyl chain at position 40, are actually widely used as immunosuppressant agents in solid organ transplant recipients. Both drugs have a narrow therapeutic index, poor correlations between the doses and blood concentrations, and therapeutic monitoring is necessary in order to minimize adverse side-effects and to ensure effective immunosuppression (1–4). As the highest proportion of sirolimus and everolimus is found within erythrocytes, EDTA-anticoagulated whole blood is the appropriate matrix for the determination of the trough concentration, which presents a good correlation with the area under the concentration-time curve (1–5). Therapeutic trough concentration ranges of 5–15 μg/L for sirolimus (1–3,6) and 3–8 μg/L for everolimus (4,7) are generally accepted.

An algorithm based on the concentration/dose ratio, using a previous trough level determination corresponding to a particular dose for the prediction of subsequent dosages of sirolimus in kidney transplant patients, has been previously proposed by Wyeth Pharmaceuticals (8). The aim of our study was the determination of the intra- and interindividual variability of sirolimus and everolimus clearance in renal and liver transplant recipients, in order to evaluate the diagnostic efficiency and clinical usefulness of the concentration/dose ratio for the prediction of steady-state trough concentrations (or dosage regimens) of both drugs.

## Patients and methods

A total of 16 patients treated with sirolimus were studied, with an age (mean ± SEM) of 53.9 ± 3.8 years (range 19–75 years), of which 7 (5 men and 2 women) were maintenance renal transplant recipients, and 9 (men) were maintenance liver transplant recipients. In 4 cases, sirolimus was administered in monotherapy, in 4 tacrolimus was associated, in 3 tacrolimus+mycophenolate, in 3 mycophenolate, in 1 cyclosporin, and in 1 cyclosporin+tacrolimus. The group treated with everolimus was comprised of 40 patients with an age of 56.7 ± 1.5 years (range 27–72 years), of which 20 (11 men and 9 women) were maintenance renal transplant patients, 17 (16 men and 1 woman) were maintenance liver transplant patients, and 3 (men) were kidney and liver recipients. In 5 cases, everolimus was administered in monotherapy, in 5 tacrolimus was associated, in 13 tacrolimus+mycophenolate, in 12 mycophenolate, in 1 cyclosporin, and in 4 cyclosporin+mycophenolate. Several blood samples from each of the different transplant patients were taken prior to the next dose of sirolimus or everolimus, and at least after a 10-day period without any modification of the dosage or concomitant administered drugs. Consequently, the blood concentrations of both immunosuppressant drugs correspond to the steady-state trough levels (Css). The study was carried out according to the good practice rules for investigation in humans of the Conselleria de Sanidade (Regional Ministry of Health) of the Xunta de Galicia, Spain.

Sirolimus determination was carried out using the IMx sirolimus microparticle enzyme immunoassay (MEIA) from Abbott Laboratories (Abbott Park, IL, USA) according to the manufacturer's specifications. The concentrations of sirolimus were also determined by high-performance liquid chromatography (HPLC) with UV detection (9). The determination of everolimus was carried out using the IMx sirolimus MEIA (Abbott Laboratories), as previously described (10). The imprecision of the methods used for the determination of both immunosuppressive agents may be considered as very satisfactory, with within- and between-run variation coefficients of 3.4%–8.6% using MEIA and 3.9%–11.9% using HPLC for sirolimus determination (9), and 2.8%–7.3% using MEIA for everolimus determination (10). Sirolimus and everolimus clearance (CL) was estimated using the equation (11): CL = (F)(D/τ )/Css, where F corresponds to the bioavailability (0.15 for sirolimus (6) and 0.16 for everolimus (7) were considered), D is the dose, τ the dosing interval, and Css the whole-blood concentration. As trough rather than mean concentrations were used, the reported CL may represent overestimates of the actual values. For the prediction of sirolimus and everolimus steady-state trough concentrations, the measured concentrations on starting the study (Css_0_), corresponding to the particular doses (D_0_), were used for the calculation of the predicted concentrations (Css) corresponding to other doses (D) using the expression: Css = (Css_0_)(D)/D_0_. The calculation of the prediction error was made using the equation: ((Css-Css_0_)/Css_0_)×100, and expressed as a percentage. Serum levels of albumin, bilirubin, creatinine, urea, alanine aminotransferase (ALT), aspartate aminotransferase (AST), cholinesterase (ChE), alkaline phosphatase (ALP), and gamma glutamyltransferase (GGT) were determined in an Advia 2400 Chemistry System (Siemens Healthcare Diagnostics Inc., Newark, DE, USA). The glomerular filtration rate (GFR) was estimated using the six-variable (age, sex, race, and serum creatinine, urea, and albumin concentrations) Modification of Diet in Renal Disease formula (12).

Statistical analysis was carried out using the StatGraphics Plus (v. 5.0) package. The Shapiro-Wilks method was used to check the distribution of data, and Pearson's correlation coefficient was used when the data had a Gaussian distribution, otherwise, Spearman's correlation coefficient was used. The regression analysis was made using the Passing-Bablock non-parametric method. In accordance with the proposed validation criteria of analytical methods for the quantitative determination of drugs and their metabolites, the acceptance criterion for accuracy is a deviation of no more than 15% from the nominal value (13,14).

## Results

**Figure 1.**
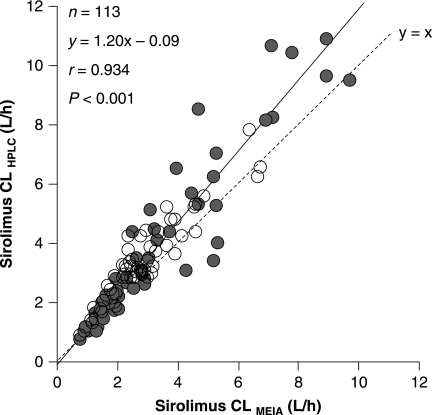
Correlation and regression between the sirolimus clearance (CL) values obtained using the blood concentrations determined by microparticle enzyme immunoassay (MEIA) and high-performance liquid chromatography (HPLC) in the kidney (○) and liver (•) transplant recipients.

Figure 1 shows the correlation and regression found between the values obtained for sirolimus CL using the blood concentrations determined by MEIA and HPLC. The correlation obtained has to be considered as highly satisfactory, although, as would be expected taking into account the sirolimus-metabolite cross-reactivity with the antibody of the immunoassay used (9), the sirolimus CL obtained from the MEIA data is significantly lower (mean 3.1 ± 0.2 L/h, median 2.6 L/h versus mean 3.6 ± 0.2 L/h, median 3.1 L/h, *P* < 0.001). The results indicated below for sirolimus CL were calculated based on the concentrations determined by MEIA; however, the use of the CL values obtained from HPLC concentrations led to the same conclusions, without providing any data of further interest.

Table I shows the results obtained for CL of sirolimus and everolimus in the groups of kidney and liver transplant patients. In the kidney transplant patients, an intraindividual variability for sirolimus CL was found of 25.6%, and an interindividual variability of 44.2%, and for everolimus CL 20.0% and 45.0%, respectively. In the liver transplant patients, sirolimus CL presented an intraindividual variability of 44.4% and an interindividual variability of 86.0%, and everolimus CL 23.0% and 40.0%, respectively. In the patients with kidney/liver transplants, an intraindividual variability for everolimus CL was found of 42.7% and an interindividual variability of 84.1%.

**Table I.** Trough concentrations (Css) and clearance (CL) of sirolimus and everolimus in kidney and liver transplant recipients.

	*n*	Kidney transplant	*n*	Liver transplant
Sirolimus:				
Css (μg/L)	52	8.1 ± 0.4 (8.4)^a^	61	7.2 ± 0.5 (6.3)^a^
CL (L/h)	52	3.0 ± 0.2 (2.8)	61	3.2 ± 0.3 (2.3)
GFR (mL/min/1.73 m^2^)	52	49.4 ± 3.0 (50.9)^b^	61	66.6 ± 4.0 (72.4)^b^
Everolimus:				
Css (μ/L)	169	4.5 ± 0.1 (4.2)	80	4.6 ± 0.2 (3.9)
CL (L/h)	169	3.7 ± 0.1 (3.8)^b^	80	3.1 ± 0.1 (2.9)^b^
GFR (mL/min/1.73 m^2^)	169	45.7 ± 1.2 (42.9)	80	42.3 ± 1.9 (45.0)

^a^*P* < 0.05. ^b^*P* < 0.01. GFR = glomerular filtration rate.

**Figure 2.**
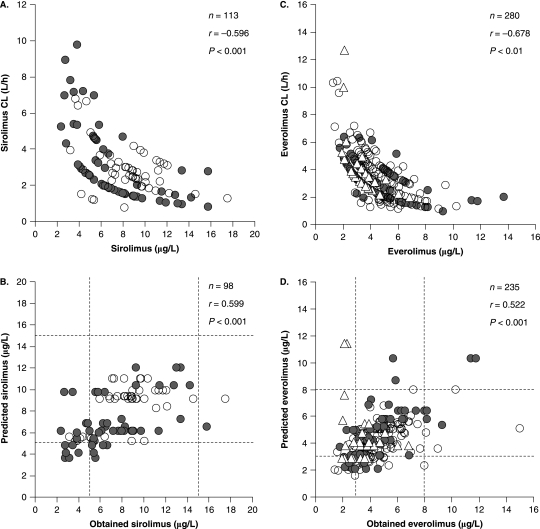
Relationship of the sirolimus and everolimus clearance (CL) with its blood concentrations (A, C), and between the predicted and obtained sirolimus and everolimus concentrations (B, D) in the kidney (○), liver (•), and kidney/liver (Δ) transplant recipients.

As shown in Figure 2, a significant negative correlation was found for the CL with the blood concentrations of sirolimus (A) and everolimus (C). Also, significant correlations were found for sirolimus CL with bilirubin (*r* = −0.210, *P* < 0.05), and for everolimus CL with bilirubin (*r* = −0.253, *P* < 0.001), AST (*r* = −0.360, *P* < 0.001), ALT (*r* = −0.283, *P* < 0.001), GGT (*r* = −0.285, *P* < 0.001), ALP (*r* = −0.217), and ChE (*r* = 0.266, *P* < 0.001). Statistical significance was not achieved in the correlation of sirolimus CL and everolimus CL with the albumin concentration. There was no significant difference in sirolimus CL (mean 2.6 ± 0.2 L/h, median 2.6 L/h versus mean 3.3 ± 0.4 L/h, median 2.8 L/h) or everolimus CL (mean 3.6 ± 0.1 L/h, median 3.6 L/h versus mean 4.4 ± 0.5 L/h, median 3.5 L/h), and sirolimus concentrations (mean 7.6 ± 0.4 μ/L, median 7.6 μg/L versus mean 8.8 ± 0.6 μg/L, median 9.3 μg/L) or everolimus (mean 4.6 ± 0.1 μg/L, median 4.2 μg/L versus mean 4.3 ± 0.3 μg/L, median 3.8 μg/L) between the patients with serum triglyceride concentrations below and above 2 mmol/L.

Figure 2 shows the correlation found between the predicted concentrations using the model indicated above and the concentrations obtained experimentally for sirolimus (B) and everolimus (D). The error (deviation) of the mean predicted concentrations with regard to those obtained, both for sirolimus (mean 7.7 ± 0.3 μg/L, median 7.5 μ/L versus mean 7.5 ± 0.2 μg/L, median 6.8 μg/L) and for everolimus (mean 4.4 ± 0.1 μg/L, median 4.0 μg/L versus mean 4.3 ± 0.1 μg/L, median 4.2 μg/L), was less than 15% and therefore acceptable according to the accuracy criterion used (13,14). However, wide ranges were obtained for the prediction error of the sirolimus and everolimus concentrations in the total group (−58.9% to 261.1% and −70.8% to 442.8%), and also in the kidney (−48.5% to 75.0% and −70.8% to 107.8%), liver (−58.9% to 261.1% and −55.0 to 114.8%), and kidney/liver (−44.1% to 442.9% for everolimus) transplant recipient groups. The mean intra- and interindividual variability of the immunosuppressive drugs CL and the prediction error obtained in the four patients treated in monotherapy with sirolimus (two renal and two liver transplant recipients) and five with everolimus (three renal and two liver transplant recipients) were only slightly smaller than those indicated above for the total of kidney and liver transplant patients.

In the total number of cases studied, the concordance in the classification as subtherapeutic, therapeutic, and supratherapeutic levels between predicted and obtained concentrations was 79.6% for sirolimus and 77.4% for everolimus. In the kidney transplant recipients this concordance was 91.3% for sirolimus and 81.4% for everolimus; however, in the liver transplant recipients the concordance was lower, 69.2% for sirolimus and 72.6% for everolimus, and in the kidney/liver transplant recipients the concordance obtained for everolimus was 67.9%.

## Discussion

Variations in sirolimus and everolimus absorption and CL result in a wide range of trough concentrations among patients receiving the same dose. The combination of cyclosporin and the two mTor inhibitors requires a reduction of both drugs due to a well-established pharmacokinetic drug interaction; however, to date no significant influence of mycophenolate on sirolimus/everolimus exposure has been reported, and with regard to the potential tacrolimus interaction, contradictory findings have been published (15).

Although intestinal absorption may not be significantly affected in hepatic dysfunction, patients with liver disease showed decreased CL of sirolimus (16,17) and everolimus (18). As shown in Table I, for sirolimus CL no significant difference was found between the groups of kidney and liver transplant patients; however, the group of kidney transplant patients had a significantly higher concentration of sirolimus (*P* < 0.05) and, in line with the results shown in Figure 2A, this could be masking a possible statistical significance of the difference between the sirolimus CL of both groups. It should also be taken into account that the group of kidney transplant patients had an estimated GFR that was significantly lower than the group of liver transplant patients (*P* < 0.001), and renal insufficiency alters intestinal, renal, and hepatic drug metabolism (19,20). A significantly higher CL was found for everolimus in the group of kidney transplant recipients (*P* < 0.001), without the presence of any significant difference between the everolimus concentrations or estimated GFR of the groups of kidney and liver transplant patients (Table I).

Recently it has been noted that serum ALP and GGT activities are quite sensitive in predicting biliary tract complications post liver transplantation, and also that AST, ALT, and bilirubin were not of any clinical significance in diagnosing these complications (21). As previously mentioned, statistically significant negative correlations were found for sirolimus CL with bilirubin and everolimus CL with bilirubin, ALP, GGT, AST, and ALT, although with modest correlation coefficients.

In *de novo* heart transplant recipients, the sirolimus apparent clearance (CL/F) was 38% lower when the triglyceride concentration was higher than 2 mmol/L (22); however, in our maintenance kidney and liver transplant patients a significant difference was not obtained for the CL of sirolimus and everolimus between the cases with triglyceride levels lower or greater than 2 mmol/L.

As shown in Figure 2 (A, C), CL values vary with sirolimus and everolimus concentration, suggesting that the metabolism of both drugs tends toward saturation as the concentration increases. However, the use of a concentration/dose ratio model to predict the dosage required to achieve a desired steady-state concentration of sirolimus from previous concentrations and corresponding doses for the same patient (8) assumes a linear relationship between sirolimus doses and concentrations (first-zero-order kinetics).

Figure 2 (B, D) shows the relationships found between the predicted concentrations and those obtained for sirolimus and everolimus. The diagnostic efficiency of a laboratory test is defined as the percentage of all results that are true, and as a general rule a test is probably not worth doing if its efficiency is less than 80% (23). As a result, the efficiency of the predictive model is acceptable in the kidney transplant recipients, with a concordance between the predicted concentrations and those obtained as subtherapeutic, therapeutic, and supratherapeutic concentrations of 91.3% for sirolimus and 81.4% for everolimus. The intraindividual variability obtained for everolimus CL was slightly lower than that of sirolimus CL, but the diagnostic efficiency was greater in the prediction of sirolimus levels due to its greater therapeutic concentration range. In any case, the wide range found for the prediction error of the sirolimus and everolimus concentrations is an important disadvantage for the routine application of this dosing model in clinical practice. In the groups of liver and kidney/liver transplant recipients, with a greater intraindividual variability for the CL values, the diagnostic efficiency found for both sirolimus and everolimus is unacceptable (<80%).

In conclusion, the dosing model using the concentration/dose ratio would have an acceptable diagnostic efficiency for the dosage adjustment of sirolimus and everolimus in kidney transplant recipients, but not in liver transplant patients. However, according to our results, the clinical usefulness of this predictive model is weak.
